# Supplementary material for: Estimating relative CWD susceptibility and disease progression in farmed white-tailed deer with rare PRNP alleles
Source: PLoS One. 2019 Dec 2;14(12):e0224342. doi: 10.1371/journal.pone.0224342 (PMC6886763; doi:10.1371/journal.pone.0224342)
Supplement: S1 Table — (DOCX) [file pone.0224342.s001.docx]

**Table S1:**

| **Genotype** | **United States** | | | **Canada** | |
| --- | --- | --- | --- | --- | --- |
|  | **Midwest**  **(N=75)** | **Northeast**  **(N=29)** | **South**  **(N=13)** | **Alberta**  **(N=4)** | **Saskatchewan**  **(N=2)** |
| **96GG** | 2087 | 849 | 199 | 297 | 276 |
| **96GS** | 1159 | 508 | 190 | 228 | 257 |
| **96SS** | 236 | 67 | 97 | 67 | 78 |
| **95H/96G** | 85 | 62 | 0 | 5 | 19 |
| **95H/96S** | 21 | 25 | 0 | 2 | 9 |
| **95HH** | 4 | 7 | 0 | 0 | 0 |
| **96G/116G** | 0 | 0 | 0 | 18 | 33 |
| **96S/116G** | 0 | 0 | 0 | 11 | 8 |
| **95H/116G** | 0 | 0 | 0 | 0 | 2 |
| **116GG** | 0 | 0 | 0 | 1 | 1 |
| **96G/226K** | 192 | 93 | 9 | 0 | 0 |
| **96S/226K** | 60 | 32 | 19 | 0 | 0 |
| **95H/226K** | 4 | 3 | 0 | 0 | 0 |
| **226KK** | 13 | 4 | 0 | 0 | 0 |
| **Total** | 3865 | 1651 | 514 | 629 | 684 |
