# Supplementary material for: Estimating relative CWD susceptibility and disease progression in farmed white-tailed deer with rare PRNP alleles
Source: PLoS One. 2019 Dec 2;14(12):e0224342. doi: 10.1371/journal.pone.0224342 (PMC6886763; doi:10.1371/journal.pone.0224342)
Supplement: Attachment — Submitted filename: PLOS One Response to criticisms.docx [file pone.0224342.s002.docx]

Public Library of Science ONE

185 Berry Street, Suite 3100

San Francisco, CA 94107 USA

Dear Dr. Caughey,

We would like to take the opportunity to thank you for your time, and that of the reviewers, in providing feedback on our manuscript entitled “Estimating relative CWD susceptibility and disease progression in farmed whitetail deer with rare PRNP alleles.” The feedback has proven very helpful in improving the manuscript, and our revisions to each comment are provided below. We first provide revisions to the Funding and Competing Interests Statements as requested:

**Funding Statement:**

Simpson Whitetails Genetic Testing provided support in the form of salaries for authors (DS and AC), as well as genotypic data from a subset of CWD negative herds, representing approximately 50% of the healthy herds reported. These authors had no role in study design or data analysis. The specific roles of these authors are further articulated in the “author contributions” section.

**Competing Interests Statement:**

Two authors (DS and AC) were employed by Simpsons Whitetails Genetic Testing at the time this study was undertaken. These two authors provide a commercial test for PRNP genotyping to the deer and elk farming industry. There are no patents or products in development associated with this commercial venture. This association does not alter our adherence to PLOS ONE policies on sharing data and materials.

Response to reviewers:

**Reviewer #1**

1. The reviewer suggests that a reference to Figure 2 be included with the statistical modelling methodology.

**Response:** We have added the reference as suggested.

2. The reviewer suggests that the qualifier “homozygous” be inserted to help clarify with genotypes are being discussed.

**Response:** We have added the qualifier “homozygous” as suggested.

3. The reviewer suggests a revision to the Tables to include information on the number of deer with each listed genotype.

**Response:** For this response, we chose to focus on Table 1, to include information on genotype and CWD status. We felt that this best addressed comments by reviewer 1 and reviewer 3. We have also included a supplemental Table S1 which presents genotype data for healthy herds (vs. allele frequency for Table 2).

4. The reviewer asks whether 116GG should have an observed outcome in Figure 2; as they astutely observed, the data was present in the manuscript.

**Response:** The modeling data for 116GG animals is available as the reviewer notes, as shown in Table 3. The relatively low number of these animals in the study led to a fairly wide error range, outside of the scale of the graphs which were practical for the Figure (-6 log vs. -4 log range for the graphs). Because of this, the observed outcome data point for these animals (which did have IHC data available) was left out of the figure for the sake of clarity. We have provided clarification in the figure legend to point out that the data is available in Table 3. As additional data is collected on these and other animals in the future, we may be able to provide further information on how well our model fit for these graphs for homozygous animals.

5. The reviewer identified a grammatical error in the legend for Figure 2.

**Response:** We have corrected this error in the final manuscript.

**Reviewer #2**

1. The reviewer notes that the common name for the species reported is “white-tailed deer.”

**Response:** We have amended all occurrences of “whitetail deer” to “white-tailed deer” throughout the manuscript.

2. The reviewer points out that data is a pleural noun, and that verbs throughout the manuscript should be corrected appropriately.

**Response:** We have corrected the errors throughout the manuscript.

Reviewer #3

1. The reviewer suggests that our analysis associates prevalence of CWD to CWD status for different genotypes, to address the influence of prevalence on disease resistance.

**Response:** The authors agree, and in fact that information is built into our modeling approach. Since this was not clear in our description of the methods, we have included clarification in our revised manuscript as follows:

“This approach accounted for differences in disease prevalence and genotypic distribution between and across farms in an effort to better estimate relative susceptibility and disease progression.”

2. The reviewer notes that we report that some genotypes were not infected on some of the higher prevalence farms, and notes the absence of data presented which specifically illustrates this.

**Response:** We agree that the tables we’ve provided do not allow the reader to fully appreciate the frequency of various genotypes present – as Reviewer 1 also suggested. In response to this, we’ve modified Table 1 to include genotype data instead of allele frequency and CWD status, and we have included a supplemental Table 1 that covers genotypic data for healthy herds.

3. The reviewer asks whether the healthy herds that were evaluated are currently importing or moving deer, and whether an entire herd was sampled for the analysis.

**Response:** Many of the healthy herds included in our analysis are importing and exporting deer, semen, and in some cases embryos. A small subset of these herds are entirely closed and self-sufficient. The analysis solely includes data from herds where all animals were available for genotyping, to avoid bias as the reviewer notes. We have made note of this in the methods section as follows: “Data from these healthy herds solely included locations where the entire herd was sampled”

4. The reviewer finally asks whether there was a significant distance between the Canadian and United States herds evaluated, which might offer some insight into the distribution of 116G and 226K alleles.

**Response:** The geographic distance between the nearest Canadian and US herds is roughly 400 miles, however the question likely goes beyond geographical distance and more likely lies in sire selection/preferences and international laws covering the movement of deer and deer products (e.g. semen). It is worth noting that historically, the 116G allele has been reported on a farmed deer herd depopulated in Nebraska (O’Rourke et al, JGV, 2004), and this herd was rumored to be owned by a Canadian deer farmer who may have moved deer between the US and Canada prior to regulations preventing international movement of cervids. We feel that it is likely that either of these currently unreported alleles may eventually be found in their respective US or Canadian herds, however it is also likely their frequencies will be quite low.

We submit the revised manuscript, included a copy with changes highlighted, for your further consideration for publication in PLOS One, and sincerely thank you for your assistance and contributions.

Sincerely yours,

Nicholas James Haley

Kahla Merrett

Amy Buros-Stein

Dennis Simpson

Andrew Carlton

Antanas Staskevicius

Tracy Nichols

Aaron Lehmkuhl

Bruce Thomsen
